# Induction of a Th17 Phenotype in Human Skin—A Mimic of Dermal Inflammatory Diseases

**DOI:** 10.3390/mps2020045

**Published:** 2019-06-04

**Authors:** Sara M. Garrett, Qihong Zhao, Carol Feghali-Bostwick

**Affiliations:** 1 Department of Medicine, Division of Rheumatology, Medical University of South Carolina (MUSC), Charleston, SC 29425, USA; smg21@musc.edu; 2 Bristol-Myers Squibb, Princeton, NJ 08543, USA; qihong.zhao@bms.com

**Keywords:** IL-17, Th17 cytokine, *ex vivo*, skin

## Abstract

Th17 cells are a subset of effector T helper cells that produce interleukin (IL)-17A, IL-17F, IL-22, and IL-26, which can promote tissue inflammation and contribute to the pathogenesis of rheumatic, fibrosing, and other diseases. Research into these diseases is often limited by a lack of an animal model that closely mimics human disease and the paucity of patient clinical tissues. Therefore, the development of relevant experimental models is crucial. Three media formulations of Th17-skewing cocktail (CT) were evaluated for the ability to induce a Th17 signature in an *ex vivo* human skin model: CT9 contained αCD3, αCD28, IL-23, IL-1β, IFNγ, IL-4, IL-6, IL-21, and TGFβ; CT8 lacked IL-1β; and CT4 only contained αCD3, αCD28, IL-23, and IL-1β. Healthy donor skin was defatted, distributed as 3 mm punch biopsies, and incubated with one of the cocktail formulations or vehicle for 48 h. All of the cocktail formulations independently significantly stimulated the expression of each gene examined. CT4 induced *IL-17A* expression 1024-fold, significantly higher than CT9 and CT8. *IL-17F* was robustly stimulated by CT4 (1557-fold), CT9 (622-fold), and CT8 (111-fold), with significant differences between the CT groups. All of the formulations significantly induced *IL-22* (16–42-fold). CT9 stimulated the highest *IL-26* response (41-fold), which was significantly higher than CT4 and CT8. *IL-10* was stimulated significantly higher with CT8 (10-fold) than CT4 or CT9. The secretion of IL-17A was significantly elevated with all cocktail formulations. Robust *IL-17A*/*IL-17F* cytokine induction was preferentially mediated by CT4, which suggested that its components are the minimal constituents necessary for the full induction of these genes in this human skin explant model, while the downstream cytokines were preferentially upregulated by CT4 (*IL-22*), CT9 (*IL-26*), or CT8 (*IL-10*). In summary, our findings suggest that the induction of a Th17 phenotype in human skin is feasible and can be used as a model for rheumatic and fibrosing diseases where Th17 skewing is observed.

## 1. Introduction

Naïve T cells can be activated and differentiated into distinct lineages, including T helper (Th) 1, Th2, and Th17 cells [1,2,3]. Th17 cells have been found to contribute to several pathologies comprising auto-immune, inflammatory, and fibrotic mechanisms, such as multiple sclerosis, psoriasis, rheumatoid arthritis, systemic lupus erythematosus (SLE), and systemic sclerosis (SSc, scleroderma) [4,5,6,7]. Mediated through the master orchestrating transcription factor retinoic acid receptor-related orphan nuclear receptor (RORγτ), Th17 cells release Th17-related cytokines, including interleukin (IL)-17A, IL-17F, IL-22, IL-26, and IL-10 [8,9,10].

IL-17A and IL-17F belong to a family of IL-17 cytokines containing six members (IL-17A–IL-17F). The effects of IL-17A are situational; in murine colitis models, IL-17A functions in a tissue-protective manner, while the pro-inflammatory role of IL-17A has been delineated in arthritis and psoriasis in other contexts [11,12,13]. IL-10, IL-22, and IL-26 belong to the IL-10 cytokine family [14]. IL-10 cytokines have been reported to be immunoregulatory, exhibiting both pro-inflammatory and anti-inflammatory effects, depending on the temporal, pathological, and spatial conditions [15,16,17].

Research into Th17-associated diseases is often limited by the lack of an animal model that closely mimics human disease and/or paucity of patient clinical tissues. *In vitro* cultures utilizing purified naïve CD4+ T cells, dendritic cells, or co-cultures are limited in that they provide responses from a select subset of cells and often incorporate sera or innate triggers from non-human species [18,19,20]. Current animal models, such as bleomycin-induced rodent fibrosis or experimental autoimmune encephalitis, often require significant time and expense [5,21]. Having a cost-effective *ex vivo* model with direct relevance to the human disease to study effects on a Th17 phenotype is essential for bolstering scientific knowledge in the field. We have previously validated an *ex vivo* human skin model for the induction of fibrosis while using multiple pro-fibrotic agents, which resulted in significant collagen cross-linking, dermal and collagen bundle thickness, and pro-fibrotic gene expression indicative of fibrosis [21,22,23], suggesting that the *ex vivo* human skin model could potentially be utilized as a model for other skin conditions. We show herein that several Th17-skewing cocktail formulations led to the upregulation and secretion of Th17 cytokines in our human *ex vivo* skin model, which can serve as an organ culture system for the study of human diseases with Th17 cytokine involvement, including SSc, psoriasis, neutrophilic dermatoses, and SLE.

## 2. Materials and Equipment

### 2.1. General Materials

- Serological pipets, various sizes (ThermoFisher Scientific, Waltham, MA, USA; Cat. nos.: 13-678-11C, 13-678-11D, 13-678-11E)
- Barrier pipet tips, various sizes (ThermoFisher Scientific, Waltham, MA, USA; Cat. nos.: 02-717-158, 02-717-161, 02-717-165, 02-717-166)

### 2.2. Materials for ex vivo Skin Culture

- Dulbecco’s modified Eagle’s medium (DMEM; MediaTech, Oceanside, CA, USA; Cat. no.: 10-013)
- Antibiotic/antimycotic (penicillin, streptomycin, and amphotericin B; ThermoFisher Scientific, Waltham, MA, USA; Cat. no.: 15-240-062)
- Sterile disposable 3 mm-diameter biopsy punches (ThermoFisher Scientific, Waltham, MA, USA; Cat. no.: 12-460-406)
- Non-pyrogenic polystyrene multi-well plates (ThermoFisher Scientific, Waltham, MA, USA; Cat. no.: 08-757-214)
- Cell culture dishes, 10 cm ((ThermoFisher Scientific, Waltham, MA, USA; Cat. no.: 07-000-386)
- Single-edge steel razor blades, individually wrapped (VWR, Radnor, PA, USA; Cat. no.: 55411-050)

### 2.3. Materials for Th17-Skewing Cocktail

- Anti-CD3 antibody (Abcam, Cambridge, MA, USA; Cat. no.: ab8090)
- Anti-CD28 antibody (R & D Systems, Minneapolis, MN, USA; Cat. no.: MAB342)
- Recombinant human IL-23 (R & D Systems, Minneapolis, MN, USA; Cat. no.: 1290-IL)
- Recombinant human IL-1β (Peprotech, Rocky Hill, NJ, USA; Cat. no.: 200-01B)
- Recombinant human IFNγ (R & D Systems, Minneapolis, MN, USA; Cat. no.: 285-IF)
- Recombinant human IL-4 (R & D Systems, Minneapolis, MN, USA; Cat. no.: 204-IL)
- Recombinant human IL-6 (R & D Systems, Minneapolis, MN, USA; Cat. no.: 206-IL)
- Recombinant human IL-21 (R & D Systems, Minneapolis, MN, USA; Cat. no.: 8879-IL)
- Recombinant human TGFβ1 (R & D Systems, Minneapolis, MN, USA; Cat. no.: 240-B)
- Sterile PBS (ThermoFisher Scientific, Waltham, MA, USA; Cat. no.: 17-516F)
- Sterile RNAse-free water (ThermoFisher Scientific, Waltham, MA, USA; Cat. no.: 10-977-015)

### 2.4. Materials for Endpoint Analyses

- TRIzol^TM^ (ThermoFisher Scientific, Waltham, MA, USA; Cat. no.: 15-596-018)
- Hard tissue homogenizing 2 mL reinforced bead tubes, nuclease-free (Omni International, Kennesaw, GA, USA; Cat. no.: 19-628)
- Sterile RNAse-free water (ThermoFisher Scientific, Waltham, MA, USA; Cat. no.: 10-977-015)
- SuperScript^TM^ IV First-Strand Synthesis system (ThermoFisher Scientific, Waltham, MA, USA; Cat. no.: 18091050)
- TaqMan^TM^ Gene Expression Master Mix (ThermoFisher Scientific, Waltham, MA, USA; Cat. no.: 4369016)
- MicroAmp^TM^ Fast Optical 96-well reaction PCR plates (ThermoFisher Scientific, Waltham, MA, USA; Cat. no.: 43-469-07)
- IL-17A primers (ThermoFisher Scientific, Waltham, MA, USA; Cat. no.: Hs00174383_m1)
- IL-17F primers (ThermoFisher Scientific, Waltham, MA, USA; Cat. no.: Hs00369400_m1)
- IL-22 primers (ThermoFisher Scientific, Waltham, MA, USA; Cat. no.: Hs01574154_m1)
- IL-26 primers (ThermoFisher Scientific, Waltham, MA, USA; Cat. no.: Hs00218189_m1)
- IL-10 primers (ThermoFisher Scientific, Waltham, MA, USA; Cat. no.: Hs00961622_m1)
- PPIB primers (ThermoFisher Scientific, Waltham, MA, USA; Cat. no.: Hs00168719_m1)
- Human IL-17A Coated ELISA kit (ThermoFisher Scientific, Waltham, MA, USA; Cat. no.: BMS2017)

### 2.5. Equipment

- Biological Safety Cabinet (Labconco Corporation, Kansas City, MO, USA; Cat. no.: 362090412242)
- Pipet-Aid^®^ (Drummond, Broomall, PA, USA; Cat. no.: 4-000-100)
- Pipettes capable of measuring 1 μL–1000 μL (Gilson, Middleton, WI, USA; Cat. no.: F167370)
- Stainless steel sharp-pointed scissors (ThermoFisher Scientific, Waltham, MA, USA; Cat. 08-940)
- Stainless steel fine point forceps (ThermoFisher Scientific, Waltham, MA, USA; Cat. 22-327379)
- Incubator (ThermoFisher Scientific, Waltham, MA, USA; Cat. no.: 13-998-211)
−80°C Ultra-Low Temperature Upright Freezer (ThermoFisher Scientific, Waltham, MA, USA; Cat. no.: 907)
- Micro Centrifuge (VWR, Radnor, PA, USA; Cat. no.: 2405-37)
- BeadRuptor24 (Omni International, Kennesaw, GA, USA; Cat. no.: 19-2141A)
- NanoDrop Lite spectrophotometer (ThermoFisher Scientific, Waltham, MA, USA; Cat. no.: ND-LITE)
- C1000 TouchTM Thermal Cycler (Bio-Rad Laboratories, Hercules, CA, USA; Cat. no.: 1851148)
- StepOne^TM^ Real-Time PCR System (ThermoFisher Scientific, Waltham, MA, USA; Cat. no.: 4376357)
- Synergy H1 microplate reader (BioTek Instruments, Inc., Winooski, VT, USA; Cat. no.: H1M)

## 3. Procedure

*Ex vivo* human skin model. Acquire human skin from healthy donors undergoing elective surgery following the rules of the Declaration of Helsinki and in accordance with guidelines set forth by the Institutional Review Board of the Medical University of South Carolina. This study is designated as non-human subject research, as the skin tissues are remnants from plastic surgery obtained without identifiers. Table 1 elaborates on the anatomical sources of donor skin.

**Figure 1:**
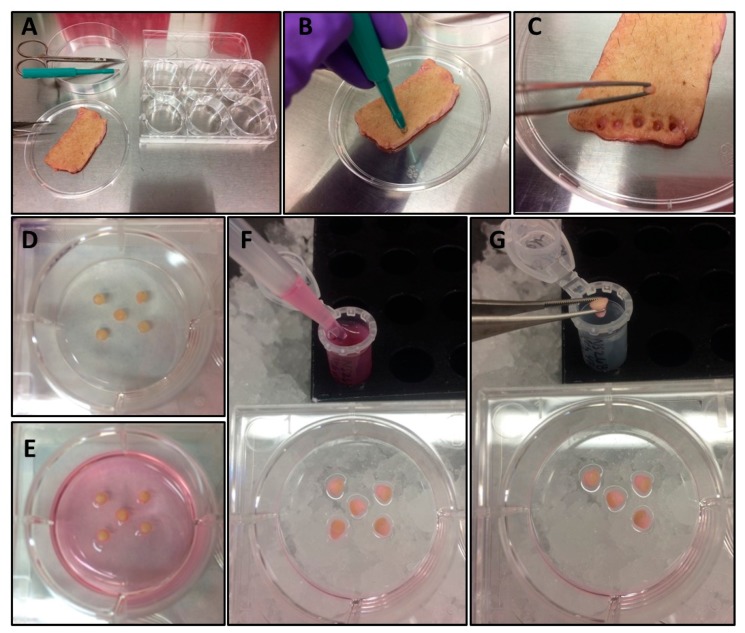
Experimental setup and sample harvest. Workflow setup (**A**), skin punching (**B**), transfer of skin punch from donor skin to treatment plate using forceps (**C**), adherence of punched skin to well (**D**), punched skin in treatment media (**E**), harvest of treatment media (**F**), and harvest of skin punch (**G**).

1. Prior to treating skin, ensure that all reagent stocks have been prepared and treatment components have been resuspended in the vehicle recommended by the manufacturer (most require PBS). The suggested stock concentrations should range between 500×–1000× whenever possible.
  1.1. Aliquot into small or single-use volumes to minimize freeze-thaw cycles and store at −80 °C until the day of the assay.
  1.2. Time for Completion: ~1 h, including labeling of aliquot tubes.
2. Defat and clean skin with ethanol and PBS, as previously described [21].
  2.1. Briefly, place skin dermal-side down atop a layer of aluminum foil on the working surface in a biological safety cabinet and clean blood/debris off of dermis using 70% ethanol.
  2.2. Separate the dermal and adipose layers using scissors and razor blade/scalpel.
    2.2.1. Discard skin portions containing stretch marks, birth marks, and other imperfections, as well as all adipose tissue.
    2.2.2. Using scissors, cut skin into pieces sized ~1–2 inches.
  2.3. Assemble five conical tubes containing ~45 mL 70% ethanol and three conical tubes containing ~45 mL sterile PBS each to clean skin.
    2.3.1. Vigorously dip a piece of skin into the first tube of ethanol 5–6 times, then repeat dipping likewise into each of the next four ethanol tubes, followed by each of the PBS tubes, in a successive manner to clean the skin.
    2.3.2. Place the cleaned skin dermal-side down in a 10 cm dish.
    2.3.3. Repeat for all pieces of skin, arranging the skin pieces in a non-overlapping manner into 10 cm dish(es).
  2.4. Add serum-free DMEM with 2× antibiotic/antimycotic to cover the bottom of the dish, leaving the top of the skin exposed to air.
  2.5. Incubate at 37 °C/5% CO_2_ until ready for use.
  2.6. Time for Completion: 30–60 min including setup and cleanup.
3. Using a 3 mm punch biopsy, punch and distribute 3–5 biopsies from cleaned, defatted skin into each well of six-well tissue culture plate to be used for the experiment (Figure 1A–D).
  3.1. Place each biopsy with the dermal side contacting the plastic and the epidermal side facing up (Figure 1D).
  3.2. Time for Completion: Time is dependent on the size of the experiment. Allot ~20 min per 6-well plate.
  3.3. Allow skin punches to adhere approximately 15 min. before adding treatment media.
4. While waiting for punches to adhere, prepare treatments as follows:
  4.1. Prepare cocktail and vehicle master mixes per Table 2 in serum-free DMEM +1% antibiotic/antimycotic.
    4.1.1. Cocktail formulations (i.e., CT4, CT8, and CT9) are named according to the number of constituent components, as follows: CT9 contains 1 μg/mL each of anti-CD3, anti-CD28, IL-4, and IFNγ, 10 ng/mL each of IL-23, IL-1β, IL-6, and IL-21, and 1 ng/mL TGFβ; CT8 contains all of the CT9 components, except IL-1β; and, CT4 only contains anti-CD3, anti-CD28, IL-23, and IL-1β.
  4.2. Whether treating a single well or more than one well with the same formulation, it is recommended to make a master mix, combining all of the components that are required in a conical tube first.
  4.3. Mix the tubes containing the cocktail component formulations, and then distribute treatments into well(s) containing the skin punches in a dropwise fashion to avoid dislodging the skin punches from the plastic (Figure 1E).
    4.3.1. For six-well tissue culture plates, a volume of 1 mL–2 mL is recommended, depending on the height of the skin punch.
    4.3.2. Add sufficient media to cover the dermal layer of skin, leaving the epidermal layer exposed to air.
      4.3.2.1. When viewed from the side of the well, the dermal layer should be completely submerged in media, leaving the topmost epidermal portion exposed to the air above the surface of the media.
  4.4. Time for completion: ~15 min.
5. Incubate at 37 °C/5% CO_2_ for 48 h.
6. Harvest skin punches and supernatants into Eppendorf tubes and freeze at −80°C until analysis (Figure 1F–G).
  6.1. Label three sets of Eppendorf tubes, one set to store skin punches and two sets for supernatants.
  6.2. Using a P1000 pipette, transfer media from each treatment well into a corresponding pre-labeled Eppendorf tube (Figure 1F).
    6.2.1. Centrifuge at 5000 RPM for 5 min to pellet cellular debris and dead cells.
    6.2.2. Transfer liquid portion (supernatant) to clean pre-labeled Eppendorf tube on ice. Discard the tube containing pellet.
  6.3. Transfer skin punches to corresponding Eppendorf tube on ice (Figure 1G).
    6.3.1. Skin punches from the same treatment well can be frozen into one Eppendorf tube. Stagger the punches along the inside wall of the tube to ease future removal once frozen.
  6.4. Freeze tubes containing skin punches and centrifuged supernatants at −80 °C.
  6.5. Time for completion: Time is dependent on the size of the experiment. Allot ~20 min per 6-well plate.
7. **RNA isolation, reverse transcription, and quantitative real-time polymerase chain reaction.**
  7.1. Homogenize one skin punch per treatment in 1 mL TRIzol^TM^ using a Bead Ruptor 24 tissue homogenizer for 30 s at a speed of 6.95, followed by two minutes on dry ice, repeated for a total of 4–5 cycles.
    7.1.1. Time for Completion: 20–30 min.
  7.2. Transfer homogenate out of bead tubes and into clean Eppendorf tubes.
    7.2.1. Time for Completion: 5 min.
  7.3. Isolate RNA using the TRIzol^TM^ separation method per the manufacturer’s instructions and resuspend in purified nuclease-free water.
    7.3.1. Time for Completion: 3 h.
  7.4. Quantify RNA and reverse-transcribe cDNA from 2 μg RNA per 20 μL cDNA reaction using the SuperScript^®^ IV First-Strand Synthesis system and oligo (dT) primers.
    7.4.1. Time for Completion: 1 h.
  7.5. In a 10 μL final reaction volume including best-coverage TaqMan^®^ Gene Expression primers, include 2 μL cDNA and plate in replicate for real-time PCR.
    7.5.1. The baseline expression of target genes tends to be undetectable after 55 cycles in vehicle-treated samples when starting from 1 μg of RNA and 1 μL of cDNA. Using greater quantities of starting material specified in this protocol helps to ensure a calculable Ct value for vehicles, which aids in quantifiable data analysis.
    7.5.2. Time for Completion: 3 h.
  7.6. Determine gene expression using the delta delta Ct method [24], normalizing data to the housekeeping gene peptidylprolyl isomerase B (PPIB).
    7.6.1. Relative fold change = 2^−ΔΔ*Ct*^ where *Ct* = threshold cycle and ΔΔ*Ct* = ((Treatment gene of interest *Ct* value − Vehicle gene of interest *Ct* value) − (Treatment PPIB *Ct* value − Vehicle PPIB *Ct* value)).
    7.6.2. Time for Completion: 1 h.
8. **IL-17A ELISA.**
  8.1. Measure the secreted IL-17A in supernatants diluted 1:1 using a commercially available sandwich ELISA kit with a dynamic detection range between 1.6 pg/mL–100 pg/mL per manufacturer’s instructions.
    8.1.1.
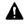
**CRITICAL STEP** Thaw supernatants on ice.
  8.2. Using a plate reader, read absorbance at 450 nm.
  8.3. When calculating IL-17A secretion, be sure to multiply values by 2 to account for the dilution that was performed according to kit instructions.
    8.3.1. *Time for Completion:* 6–7 h.

## 4. Expected Results

**Figure 2:**
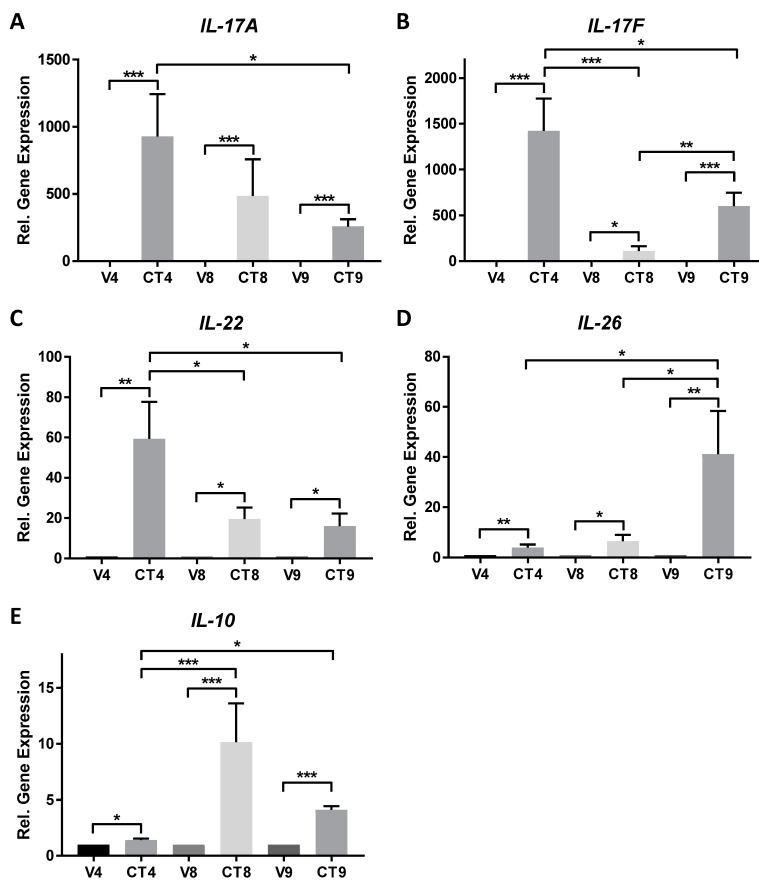
Expression of Th17-related cytokines in an *ex vivo* human skin explant model stimulated with 4-component (CT4), 8-component (CT8), or 9-component (CT9) Th17-skewing cocktail or matched vehicle for 48 h. Gene expression of *IL-17A* (**A**) with *n* = 7–16 unique donors; *IL-17F* (**B**) with *n* = 8–24 unique donors; *IL-22* (**C**) with *n* = 5–16 unique donors; *IL-26* (**D**) with *n* = 5–17 unique donors; and, *IL-10* (**E**) with *n* = 6–19 unique donors. Samples were tested in technical replicates of 2–3 and averages used to determine fold change of gene expression relative to appropriate vehicle using the delta delta Ct method and graphed in histograms as mean ± standard error of the mean. * *p* < 0.05, ** *p* < 0.01, and *** *p* < 0.001 using ordinary one-way ANOVA with Tukey’s multiple comparison test.

**Figure 3:**
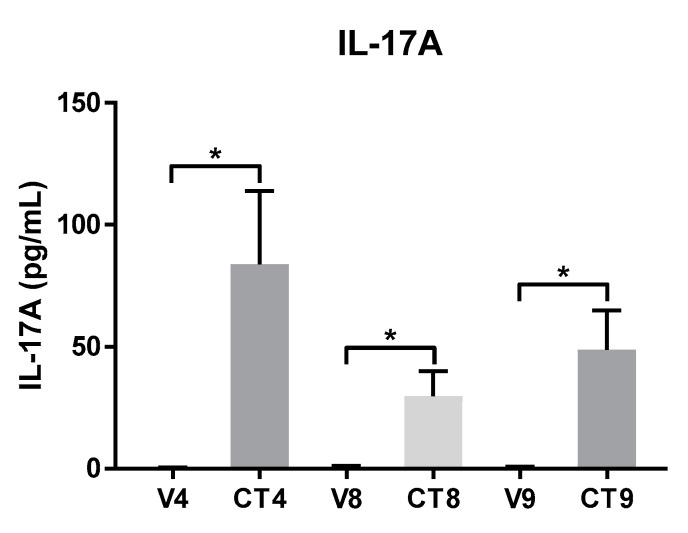
Secretion of IL-17A protein from an *ex vivo* human skin explant model stimulated with 4-component (CT4), 8-component (CT8), or 9-component (CT9) Th17-skewing cocktail or matched vehicle. Protein expression of IL-17A measured from 48 h supernatants in duplicate using a commercially available ELISA kit is graphed as a histogram showing mean ± standard error of the mean. * *p* < 0.05, ** *p* < 0.01, and *** *p* < 0.001 using ordinary one-way ANOVA with Tukey’s multiple comparison test.

Three Th17-skewing cocktail formulations were scrutinized for their ability to stimulate skin-resident cells into producing Th17-related cytokines. Cocktail components were identified from a search of the literature and formulations were designed in order to determine the minimal components that are necessary to elicit maximal gene expression of Th17-related cytokines in this *ex vivo* model. The graphs in Figure 2 and Figure 3 represent mean values +/− standard errors of the mean. Statistical significance of *p* < 0.05 utilizing analysis of variance with Tukey’s multiple comparison test with significance is denoted as * *p* < 0.05, ** *p* < 0.01, and *** *p* < 0.001. The following descriptions represent the expected results and they include data obtained over time and across multiple anatomical skin locations. The lack of significant stimulation of gene or protein expression could indicate failure of stimulation, most likely either due to a non-responsive donor tissue or to expired component(s) in the cocktail formulations.

### 4.1. Regulation of Gene Expression by Different Cocktail Formulations

*IL-17A* is significantly induced by CT4 (1024-fold), CT8 (552-fold), and CT9 (270-fold) as compared to their respective vehicle controls, with CT4 induction of *IL-17A* approximately twice that of CT8 and 3.8-times that of CT9 (Figure 2A). 

*IL-17F* expression is robustly induced by CT4 (1557-fold), CT9 (622-fold), and CT8 (111-fold), with significant differences between each of the CT groups (Figure 2B). The induction of *IL-17F* by CT4 is 2.5-times higher than CT9 and 14-times that of CT8. 

*IL-22* expression follows the same trend as *IL-17A* with respect to Th17-skewing cocktail stimulation (Figure 2C), though with a more modest induction ranging from 16-fold (CT9) to 42-fold (CT4). CT8 induction of *IL-22* is similar to that of CT9 (~20-fold), and there is a significant difference between CT4 and the other two groups. 

CT9 stimulates the highest *IL-26* response (41-fold), which is significantly higher than the other two formulations (Figure 2D). CT4 increases *IL-26* ~5-fold above vehicle and CT8 increases it ~7-fold above vehicle. 

Gene expression levels of *IL-10* are more modest in comparison to the other genes examined (Figure 2E). *IL-10* also exhibits a unique stimulation profile in that CT8 elicits the highest expression level of the gene (10-fold), followed by CT9 (4-fold), and a very modest yet significant effect with CT4 (1.4-fold). 

### 4.2. Regulation of Protein Levels of IL-17A by Different Cocktail Formulations

The IL-17A protein levels are measured as a proof of concept in matching 48 h-treated supernatants by ELISA to assess whether the induction of gene expression correlates with the increased production of the corresponding protein (Figure 3). The absolute amount of IL-17A protein released by cocktail stimulation is significantly higher than by the respective vehicle in all three formulations; however, there is not a statistically significant difference between the cocktail groups. On average, CT4 led to the secretion of 84 pg/mL IL-17A, CT8 causes 30 pg/mL IL-17A to be secreted, and CT9 causes an intermediate amount of IL-17A to be secreted (49 pg/mL) into media conditioned by the tissue cores following 48 h stimulation.

### 4.3. Interpretation/Discussion:

Various stimuli have been reported to induce Th17 cell development and promote Th17-related cytokine release. IL-23 has consistently been found to be crucial for *in vivo* Th17 cell development, but is itself (without other cytokines) insufficient for Th17 stimulation; likewise, IL-6 alone cannot elicit an increase in IL-17A expression in naïve CD4+ T cells *in vitro* [25,26]. Further research has determined that Th17 cell differentiation can occur through the combined effects of the cytokines IL-1β, IL-6, and IL-23 in the absence [25] or presence [10] of TGFβ. Other groups have determined, through murine studies, that naïve T cells can be differentiated into Th17 cells through only IL-6 and TGFβ or with the addition of IL-23 [1,27]. Another study on the contribution of TGFβ and IL-6 in Th17 cell formation led a different group to determine that these factors are critical to Th17 lineage commitment in mice, but that IL-23 and IL-1β or IL-1β and IL-6 are necessary for Th17 development from naïve T cells in humans [20,25]. While some studies have shown that the initial TGFβ/IL-1β signal mediating Th17 differentiation can also cause IL-10 induction [28], others have shown that IL-1β can inhibit IL-10 production by purified Th17 cell populations *in vitro* and *in vivo* [29]. Alternatively, IL-10 production could indicate a shift from Th17 cells to regulatory T cells (Tregs) [28]. Differences in components deemed to be necessary and sufficient to elicit the secretion of Th17 cytokines can be explained by gathering results from multiple sources across species, from purified systems utilizing naïve CD4+ T cells to whole body animal models. Thus, Th17 development seems to require a combination of cytokines and factors, regardless of the *in vitro* or *in vivo* model system. Our *ex vivo* skin model bridges the gap between *in vitro* naïve T cell cultures and *in vivo* murine experiments, and extends relevance of the findings to human tissues and disease. We have shown herein that Th17 cytokines can be induced in an *ex vivo* skin model using three different combinations of cocktails, containing a variety of cytokines that are known to be essential for Th17 cell development. 

Each of the Th17-skewing cocktail formulations induced the gene expression of *IL-17A*, *IL-17F*, *IL-22*, *IL-26*, and *IL-10*, as well as the secretion of IL-17A. Though there was no clear cocktail formulation that resulted in the highest induction of all genes tested, CT4 stimulated the highest responses in three out of the five genes, as well as the highest secretion of IL-17A. CT9 clearly induced *IL-26* over that of the other formulations, while CT8 produced the greatest response in *IL-10* stimulation (which may be due to exclusion of IL-1β from CT8 or may indicate more of a shift towards Treg formation). Though the differences between the CT groups in IL-17A secretion were non-significant, the pattern of the absolute IL-17A secreted matched that of *IL-17F* gene expression (CT4 > CT9 > CT8), rather than that of *IL-17A* (CT4 > CT8 > CT9). This apparent mismatch in patterning can be explained by several factors. Gene expression data represents the transcription of *IL-17A*, whereas the protein measurement reflects its secretion, and therefore additional time may be necessary to see optimal protein secretion. Moreover, *IL-17A* RNA and protein discordance could be due to several other factors at the post-transcriptional and/or post-translational level(s), including RNA-mediated decay, translation inhibition, translation into non-functional protein, protein degradation, and epigenetic modification [30,31,32]. *IL-17A* and *IL-17F* are located immediately adjacent to each other (3’ end to 3’ end) on chromosome 6p12.2 and due to their neighboring proximity, may be co-regulated [33,34]. Additionally, IL-17A and IL-17F, while being able to form individual homodimers, can heterodimerize with each other to bind target receptors [11,35]. The ELISA assay does not distinguish between IL-17A homodimers and IL-17A/IL-17F heterodimers, thus the results may have been influenced by the presence of IL-17F in the supernatants and they ultimately represent both populations of IL-17A homodimers and IL-17A/IL-17F heterodimers. 

Other models utilizing different Th17-skewing cytokines have been previously described, including the extensive use of purified naïve T cell cultures [1,3,10,20,28]. Furthermore, investigators have generated Th17 cytokine-expressing dendritic cells or Langerhans’ cells from peripheral blood mononuclear cells through treatment with GM-CSF and IL-4 (±TGFβ), followed by a proinflammatory cytokine cocktail of IL-1β, IL-6, PGE2, and TNFα for eight days [18], a cocktail of IL-4, GM-CSF, TNFα, and PGE2, or a cocktail of IL-13, GM-CSF, IFNγ, and a TLR2/4 agonist from *Klebsiella pneumoniae* [19]. The limitations for these models include the fact that they are *in vitro* models representing responses primarily from one cell type and methodology that includes incubation with non-human sera. A more complex model, deemed the skin resident immune cell activation assay (sRICA), in which skin is microtomed to a depth of 750 μm, includes incubation with bovine collagen, bovine serum, and cornification medium, followed by a Th17-skewing cocktail of CD3, CD28, IL-1β, IFNγ, IL-4, IL-6, IL-21, and TGFβ, similar to our CT9, but lacking IL-23 [36]. Our system addresses some of the limitations of the sRICA assay, including the incorporation of different combinations of Th17-skewing cytokines, cleaner experimental system without additional bovine collagen or serum added, and a more comprehensive system representing a larger portion of reactive skin depth, including the entirety of both the epidermal and dermal layers. When considering that the normal epidermal thickness is approximately 100 μm according to a recent systematic and comprehensive review of skin microanatomy [37], the sRICA system likely incorporates all of the epidermis and some portion of the underlying dermal layer. Since the thickness of the epidermis can be affected by skin pigmentation, smoking, vascularization, and sex [38], only a portion of the dermal contribution of fibroblasts, macrophages, other resident cells, and matrix components is captured in the sRICA assay. Furthermore, our system does not include a microtoming step that truncates the dermis, rather it incorporates a larger dermal contribution overall and allows for maintaining the skin in an air-liquid interphase with the epidermis exposed to the air, providing a comprehensive skin experimental system that more closely mimics that of living systems and that can easily translate into clinical applications of drug discovery. Therefore, our *ex vivo* Th17-skewing cocktail methodology has direct relevance to human dermal disease research that incorporates Th17 cytokine involvement, including but not limited to SSc, psoriasis, neutrophilic dermatoses, and SLE [5]. As Th17 cytokines have been identified to promote the disruption of the respiratory epithelial barrier leading to mucosal leakage [39], this same methodology could be potentially applied to an *ex vivo* lung explant model to study lung diseases with Th17 cytokine involvement.

The purpose of this model was to analyze gene expression output following Th17-skewing cocktail addition to human skin explants; as such, we did not determine the cellular source(s) of the cytokines. IL-17A, as well as the other Th17 cytokines, can be produced and secreted by a variety of cell types, which may differ between model systems, physiological locations, and across temporal expanses. Th17 cells are defined by the expression of ROR transcription factors and secretion of Th17 cytokines [10,11]. Non-Th17 sources of Th17 cytokines have been described and include CD4+ T cells, neutrophils, Paneth cells, Tregs (Foxp3+ and Foxp3-), Foxp3+IL-17A T cells, rTh17 cells (Foxp3-IL-17A+IL-10+ Th cells), γδ T cells, CD8+ T cells (TC17, TNC17), macrophages, natural killer cells, mast cells, neutrophils, polymorphonuclear cells, keratinocytes, and fibroblasts, though more sources are being regularly described [11,40,41,42,43,44,45,46].

In summary, using any of the three Th17-skewing cocktail formulations resulted in the upregulation and secretion of Th17 cytokines. Since each of the cocktail formulations uniquely performed when considering the gene expression output, they can be selectively utilized to study various diseases manifesting with unique gene expression profiles. Using the human skin in organ culture provides a model for human diseases where Th17 cells and their cytokines are implicated in disease pathogenesis, and it is a valuable tool for assessing therapies that target Th17 cells/Th17 cytokines.

## Figures and Tables

**Table 1:** Location of donor skin: #/total # (%).

Abdominal	Breast	Glutes	Arms	Multiple Sources
12/24 (50%)	8/24 (33.3%)	1/24 (4.2%)	1/24 (4.2%)	2/24 (8.3%)

**Table 2:** Cocktail Component Formulations.

Component (Final Concentration)	Cocktail 4	Cocktail 8	Cocktail 9
αCD3 (1 μg/mL)	√	√	√
αCD28 (1 μg/mL)	√	√	√
IL-23 (10 ng/mL)	√	√	√
IL-1β (10 ng/mL)	√		√
IFNγ (1 μg/mL)		√	√
IL-4 (1 μg/mL)		√	√
IL-6 (10 ng/mL)		√	√
IL-21 (10 ng/mL)		√	√
TGFβ (1 ng/mL)		√	√
